# Increased cell-free DNA in CSF and serum of hip fracture patients with delirium

**DOI:** 10.1093/braincomms/fcae452

**Published:** 2024-12-16

**Authors:** Vibeke Bratseth, Leiv Otto Watne, Bjørn Erik Neerland, Nathalie Bodd Halaas, Christian Thomas Pollmann, Adi Karabeg, Olav Tobias Odegaard, Kristian Sydnes, Henrik Zetterberg, Ingebjørg Seljeflot, Ragnhild Helseth

**Affiliations:** Oslo Center for Clinical Heart Research, Department of Cardiology Ullevaal, Oslo University Hospital, Oslo 0424, Norway; Oslo Delirium Research Group, Department of Geriatric Medicine, Oslo University Hospital, Oslo 0424, Norway; Department of Geriatric Medicine, Akershus University Hospital, Lørenskog 1478, Norway; Faculty of Medicine, Institute of Clinical Medicine, University of Oslo, Oslo 0318, Norway; Oslo Delirium Research Group, Department of Geriatric Medicine, Oslo University Hospital, Oslo 0424, Norway; Oslo Delirium Research Group, Department of Geriatric Medicine, Oslo University Hospital, Oslo 0424, Norway; Department of Orthopedic Surgery, Akershus University Hospital, Lørenskog 1478, Norway; Department of Orthopedic Surgery, Akershus University Hospital, Kongsvinger 2381, Norway; Department of Anesthesiology, Akershus University Hospital, Kongsvinger 2381, Norway; Department of Orthopedic Surgery, Diakonhjemmet Hospital, Oslo 0319, Norway; Institute of Neuroscience and Physiology, the Sahlgrenska Academy at University of Gothenburg, Mölndal 40530, Sweden; Clinical Neurochemistry Laboratory, Sahlgrenska University Hospital, Mölndal 40530, Sweden; Department of Neurodegenerative Disease, UCL Institute of Neurology, London WC1H9BT, UK; UK Dementia Research Institute at UCL, London WC1H9BT, UK; Hong Center for Neurodegenerative Diseases, Hong Kong HKG, China; Wisconsin Alzheimer’s Disease Research Center, University of Wisconsin School of Medicine and Public Health, University of Wisconsin-Madison, Madison, WI 53707, USA; Oslo Center for Clinical Heart Research, Department of Cardiology Ullevaal, Oslo University Hospital, Oslo 0424, Norway; Faculty of Medicine, Institute of Clinical Medicine, University of Oslo, Oslo 0318, Norway; Oslo Center for Clinical Heart Research, Department of Cardiology Ullevaal, Oslo University Hospital, Oslo 0424, Norway; Faculty of Medicine, Institute of Clinical Medicine, University of Oslo, Oslo 0318, Norway

**Keywords:** cell-free DNA, neutrophil extracellular traps, neuroinflammation, delirium, neurofilament light chain

## Abstract

Delirium is a neuropsychiatric syndrome commonly presenting during acute illness. The pathophysiology of delirium is unknown, but neuroinflammation is suggested to play a role. In this cross-sectional study, we aimed to investigate whether cell-free DNA and markers of neutrophil extracellular traps in serum and CSF were associated with delirium and neuronal damage, assessed by neurofilament light chain. Hip fracture patients (*n* = 491) with a median (25, 75 percentiles) age of 83 (74, 88) years and 69% females were enrolled at Oslo University Hospital, Diakonhjemmet Hospital, Akershus University Hospital and Bærum Hospital. Delirium was assessed daily, pre- and postoperatively. Cognitively healthy adults (*n* = 32) with a median (25, 75 percentiles) age of 75 (70, 77) years and 53% females were included as controls. Cell-free DNA was measured by using the fluorescent nucleic acid stain Quant-iT PicoGreen® in serum and CSF. Myeloperoxidase-DNA and citrullinated histone H3 were analysed by enzyme-linked immunosorbent assay in serum. Hip fracture patients have significantly higher levels of cell-free DNA and neutrophil extracellular traps in blood than cognitively healthy controls. In hip fracture patients without dementia, cell-free DNA in CSF and serum was significantly higher in patients with (*n* = 68) versus without (*n* = 221) delirium after adjusting for age and sex (70 (59, 84) versus 62 (53, 77) ng/ml, *P* = 0.037) and 601 (504, 684) versus 508 (458, 572) ng/ml, *P* = 0.007, respectively). In the total hip fracture cohort, CSF levels of cell-free DNA and neurofilament light chain were significantly correlated after adjusting for age and sex (*r* = 0.441, *P* < 0.001). The correlation was stronger in those with delirium (*r* = 0.468, *P* < 0.001) and strongest in delirious patients without dementia (*r* = 0.765, *P* = 0.045). In delirious patients without dementia, significantly higher levels of cell-free DNA in CSF and serum were shown. The association between cell-free DNA and neurofilament light chain suggest simultaneous release of cell-free DNA and neuronal damage during delirium.

## Teaser

Delirium is an acute and serious mental disorder with unknown pathophysiology. Bratseth and Watne et al. explored cell-free DNA and neutrophil extracellular traps in blood and CSF of hip fracture patients, with and without delirium, and cognitively healthy controls. They showed that cell-free DNA associated significantly with delirium and neuronal damage.

## Graphical Abstract

**Graphical Abstract:**
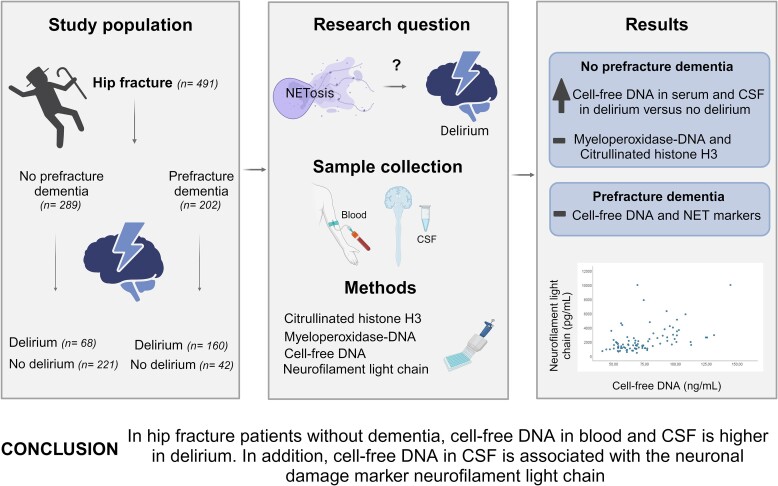

## Introduction

Delirium is a complex neuropsychiatric syndrome characterized by acute deterioration in consciousness and deficits in attention and cognitive function. Precipitating factors include advanced age, general comorbidity, severe somatic illness, surgery, infection and drug administration.^1^ The overall prevalence in hospitalized patients is approximately 20%.^2^ Delirium is associated with prolonged hospital stays, risk of future dementia and increased mortality.^3^ The strongest risk factor for delirium is cognitive impairment, and these two entities may share some common pathophysiological features.^4^

Inflammation in the CNS, i.e. neuroinflammation, seems to be associated with systemic inflammation and is believed to play a central role in the pathophysiology of delirium.^5^ The neuroinflammatory hypothesis of delirium suggests that immune-mediators and cells may cross a dysfunctional blood–brain barrier (BBB) and accelerate the inflammatory response.^5^ Higher levels of circulating interleukin (IL)-1, IL-6 and IL-8 have been shown in patients with delirium,^6,7^ and the neutrophil-lymphocyte ratio has been suggested as a potential biomarker for the early detection of delirium.^8^ Furthermore, associations between CSF levels of inflammatory mediators (for instance, IL-8 and CRP), delirium onset and underlying cognitive impairment have previously been demonstrated.^9,10^

Activated neutrophils may expel parts of their nuclear and protein content to the extracellular space in the form of neutrophil extracellular traps (NETs). This process is called NETosis. NETs are net-like structures of decondensed chromatin coated with citrullinated histones and cytotoxic proteases such as myeloperoxidase (MPO) and neutrophil elastase from the neutrophil cytosol and cytoplasmic granules.^11,12^ Citrullinated histone H3 (CitH_3_), complexes of MPO and cell-free DNA (MPO-DNA), and cell-free DNA are surrogate markers of in vivo NET formation. Whereas CitH_3_ and MPO-DNA are considered quite specific for NETosis,^13^ cell-free DNA may be released through other processes, like necrosis, apoptosis^14^ and active secretion^15^ in both blood and CSF.

Neutrophils and NETs have recently been studied in a range of diseases, where they seem to exacerbate inflammation, cause direct cell and tissue damage and promote thrombosis.^16-19^ In response to inflammatory changes in the CNS, neutrophils may migrate across the BBB into the brain parenchyma. In mouse models with and without Alzheimer's disease, it has been shown that neutrophils acquire a neurotoxic phenotype, release NETs and induce neuronal death.^20^ Neutrophil depletion or blockade of neutrophil recruitment has been shown to improve cognitive performance in mice with Alzheimer's disease.^21^ In humans, there are reports indicating a role of neutrophils and NETs in neurodegenerative diseases such as Alzheimer's disease.^22,23^ To our knowledge, there are no studies on NETs in patients with delirium.

A better understanding of the pathophysiology underlying delirium is of utmost importance for developing new treatment options. Although never studied in delirium, there are plausible reasons why NETs could play a role. First, NETs could link systemic inflammation to neuroinflammation, since neutrophils increase in number, influence BBB permeability and may infiltrate the brain during normal aging and certain pathological conditions.^24,25^ Secondly, since NETs are associated with Alzheimer's disease, it is important to explore NETs also in delirium, as these conditions are clinically linked. We hypothesized that higher levels of NET markers in the CSF and peripheral circulation were associated with delirium. As dementia is a strong risk factor for delirium,^21-23^ we also analysed our data stratified by dementia status. Neurofilament light chain (NfL) is a structural filament protein, exclusively expressed in neurons and highly specific for neuroaxonal damage.^26-29^ We assessed the relationship between cell-free DNA and NfL in CSF.

## Materials and methods

The study was performed in accordance with the Declaration of Helsinki and Good Clinical Practice procedures and approved by the Regional Committee for Ethics in Medical and health research in Norway (2009/450, 2011/2052 and 2016/1368). All participants or their proxies signed a written informed consent to participate.

### Study populations

#### Hip fracture patients

The current study population originated from two Norwegian hip fracture cohorts. The first cohort (*n* = 96) was a single-centre study with patients admitted to Oslo University Hospital between 2009 and 2012. The second cohort (*n* = 395) was a multicentre study with patients enrolled at Oslo University Hospital, Diakonhjemmet Hospital, Akershus University Hospital and Bærum Hospital, between 2016 and 2021. In both cohorts, delirium was assessed daily by trained investigators, preoperatively and until the fifth post-operative day. The delirium diagnosis was based on interviews with the patients, supplemented by information from relatives, nurses and clinical notes, as thoroughly described earlier.^30^ The Confusion Assessment Method (CAM)^31^ was used to evaluate delirium in the first cohort and the Diagnostic and Statistical Manual of Mental Disorders 5 (DSM-5) criteria for the second cohort.^32^ In both cohorts, two experienced delirium researchers (LOW and BEN) independently assessed all available information for each patient to decide whether criteria for delirium were fulfilled or not.

Patients were excluded if the hip fracture was a part of a high energy trauma (defined as an impact greater than fall from higher than 1 m) or if they were moribund on admission.

Patients were classified depending on their delirium status at the time of CSF sampling, i.e. (i) prevalent delirium: those with delirium at the time of CSF sampling (all patients with pre-operative delirium remained delirious at least the first day post-operative), (ii) incident delirium: those free from delirium at the time of CSF sampling, but who developed it later and (iii) sub-syndromal delirium (SSD): clinically defined to be in between the no delirium and delirium subgroups (i.e. patients not fulfilling all, but some, criteria for delirium). The patients with SSD were included in the no delirium group in the main analysis (delirium versus no delirium).

Prefracture cognitive status was assessed using the Informant Questionnaire on Cognitive Decline in the Elderly with ≥3.44 as a cutoff indicating cognitive impairment.^33^ In the case of missing Informant Questionnaire on Cognitive Decline in the Elderly (*n* = 29), the prefracture cognitive status was established using hospital records. The American Society of Anesthesiologists Physical Status (ASA)^34^ classification level was registered from participants’ medical records.

#### Cognitively healthy controls

Cognitively healthy controls aged >65 years were recruited from the COGNORM-study.^35^ This study included 172 participants admitted for elective surgery to Oslo University Hospital and Diakonhjemmet Hospital, Oslo during 2012 and 2013, as described previously.^35^ The participants were cognitively tested at annual follow-ups and were asked to volunteer for a second lumbar puncture with collection of CSF at the 4-year follow-up. Those with available CSF (*n* = 32) from the 4-year follow-up were included as a control group in the present work. All had a Mini Mental State Examination > 28 at baseline and showed no signs of cognitive decline the first 5 years after inclusion (exclusion criteria).^36^

### Laboratory methods

In hip fracture patients, CSF was collected in polypropylene tubes before administration of the spinal anaesthetic agent. CSF samples were centrifuged, divided into aliquots and stored at −80°C until analysis. Blood samples were collected by venous puncture at the time of CSF sampling and allowed to stand for 30 min at room temperature to clot before being centrifuged at 2200×g for 10 min. Serum was divided into aliquots and stored in polypropylene tubes at −80°C.

Levels of cell-free DNA in CSF and serum were quantified using the fluorescent nucleic acid stain, Quant-iT PicoGreen® (Invitrogen Ltd., Paisley, UK) and a fluorimeter (Fluoroskan Ascent®, Thermo Fisher Scientific Oy, Vantaa, Finland).

MPO-DNA complexes were measured in serum and CSF employing an enzyme-linked immunosorbent assay technique, previously described by Kessenbrock *et al*.^37^ Briefly, micro well plates were coated with capture antibody (anti-MPO, AbD Serotec, Hercules, CA, USA) overnight at 4°C and blocked with bovine serum albumin. Then, the patient sample and peroxidase-labelled anti-DNA antibody (Cell Death Detection kit, Roche Diagnostics GmbH, Mannheim, Germany) were added and incubated for 2 h on a shaker at room temperature. Finally, a peroxidase substrate was introduced into the wells, and the absorbance was measured after 40 min. The results are expressed as optical density.

CitH_3_ was measured in serum and CSF by a sandwich enzyme-linked immunosorbent assay kit from Cayman Chemical, Ann Arbor, USA. The assay has a lower limit of quantification of 0.3 ng/ml and lower limit of detection of 0.1 ng/ml. The intra- and inter-assay coefficients of variation (CV) for cell-free DNA, MPO-DNA and CitH_3_ in our laboratory were 1.5% and 4.6%, 13.6% and 5.4% and 4.1% and 11.4%, respectively.

CSF NfL concentrations were measured in the first hip fracture cohort using a commercial enzyme-linked immunosorbent assay (Uman Diagnostics, Umeå, Sweden), as previously described.^38^ The intra-assay CV was ≤10%.

### Statistical analysis

Statistical analyses were performed by IBM SPSS software version 26–29 (SPSS Inc., Chicago, IL, USA). The figures were made in Graph Pad prism versions 8 and 9, and the graphical abstract using BioRender. The data were not normally distributed; thus mostly non-parametric statistics were used. As participants with SSD can be classified as either cases or controls,^39^ SSD was included in the no delirium groups in the main analyses; however, sensitivity analyses were performed with SSD excluded. The data are presented as medians (25th, 75th percentiles) and proportions (%) as appropriate. Group comparisons for continuous data were performed using the Mann–Whitney U-test or the one-way ANOVA with Tukey's Honestly Significant Difference for *post hoc* comparisons of pairwise data. The ANOVA had non-significant results on the tests of homogeneity of variances (*P* > 0.5). To compare proportions and for correlation analyses, the chi-squared test of independence and Spearman's rho were assessed. A binary multivariate logistic regression model and partial rank correlation were applied to adjust for covariates (age and sex), known to influence the results. Due to the explorative study design, corrections for multiple comparisons were not performed. *P*-values <0.05 were considered statistically significant.

## Results

### Baseline characteristics

CSF samples were available from 491 hip fracture patients and 32 cognitively healthy controls. Serum samples were available from 135 hip fracture patients and 26 controls (see Supplementary Table 1 for an overview). Baseline characteristics for the respective study populations are shown in Table 1. The hip fracture patients were significantly older than the cognitively healthy controls, and the hip fracture patients with versus without delirium had a significantly higher ASA score.

**Table 1:** Baseline characteristics in hip fracture patients according to delirium status and in cognitively healthy controls

Characteristics	Hip fracture patients (*n* = 491)				Cognitively healthy controls (*n* = 32)	
	Delirium (*n* = 228)	No delirium (*n* = 263)	p_1_-value	All		p_2_-value
Age	86 (80, 91)	78 (70, 86)	**<0.001**	83 (74, 88)	75 (70, 77)	**<0.001**
Female, *n* (%)	158 (69)	181 (69)	0.987	339 (69)	17 (53)	0.094
IQCODE score ≥ 3.44, n (%)	160 (70)	42 (16)	**<0.001**	202 (41)		
ASA score^a^	3 (2, 3)	2 (2, 3)	**<0.001**			

Values are median (25, 75 percentiles) if not otherwise stated. The p_1_-value refers to group differences between delirium and no delirium, and the p_2_-value between *all* hip fracture patients and the cognitively healthy control group (Mann–Whitney U-test and chi square test of independence). Significant *P*-values are highlighted with boldface.

IQCODE; Informant Questionnaire on Cognitive Decline in the Elderly, ASA; The American Society of Anesthesiologists.

^a^The total number of hip fracture patients with an ASA score was 422 (with delirium, *n* = 199 and without delirium, *n* = 223).

### NETosis markers in serum and cell-free DNA in CSF of study populations

Hip fracture patients had significantly higher serum concentrations of cell-free DNA, MPO-DNA and CitH_3_ (all *P* < 0.01), and numerically higher cell-free DNA in CSF (*P* = 0.063) compared to cognitively healthy controls (Table 2). In hip fracture patients, cell-free DNA in CSF correlated significantly with age (*r* = 0.194, *P* < 0.001) and was significantly higher in males than in females [72 (53, 95) ng/ml versus 65 (57, 81) ng/ml, *P* = 0.002].

**Table 2:** NET markers in hip fracture patients according to delirium status and in cognitively healthy controls

	Hip fracture patients (*n* = 491)				Cognitively healthy controls (*n* = 32)	
	Delirium (*n* = 228)	No delirium (*n* = 263)	p_1_-value	All		p_2_-value
Cell-free DNA (CSF) (ng/ml)	69 (57, 88)	64 (53, 79)	**0.002**	68 (54, 84)	63 (51, 71)	0.063
Cell-free DNA (serum^a^) (ng/ml)	587 (515, 652)	511 (460, 575)	**<0.001**	547 (486, 620)	451 (421, 494)	**<0.001**
MPO-DNA (serum^a^) (OD)	0.255 (0.199, 0.381)	0.210 (0.159, 0.298)	0.054	0.230 (0.168, 0.345)	0.172 (0.139, 0.219)	**0.002**
CitH_3_ (serum^a^) (ng/ml)	11.48 (6.53, 19.47)	8.41 (4.91, 15.97)	0.087	10.50 (5.56, 18.49)	4.46 (1.92, 7.58)	**<0.001**

Values are median (25, 75 percentiles). The p_1_-value refers to group differences between delirium and no delirium, and the p_2_-value refers to group differences between *all* hip fracture patients and the cognitively healthy control group (Mann–Whitney U-test). Significant *P*-values are highlighted with boldface.

NET; neutrophil extracellular trap, MPO; myeloperoxidase, CitH_3_; citrullinated histone H_3_, OD; optical density.

^a^Serum was available in 135 hip fracture patients and 26 cognitively healthy controls.

### Cell-free DNA in serum and CSF of delirious hip fracture patients

Among the hip fracture patients, 228 experienced delirium. Patients with delirium were older (median age 86 versus 78 years, *P* < 0.001), suffered more from dementia (70% versus 16%, *P* < 0.001) and had higher levels of cell-free DNA in CSF (69 versus 64 ng/ml, *P* = 0.002) and serum (587 versus 511 ng/ml, *P* < 0.001) than the patients without delirium. Levels of MPO-DNA and CitH_3_ were not significantly higher in delirium, only numerically [(0.255 versus 0.210 optical density, *P* = 0.054) and (11.48 versus 8.41 ng/ml, *P* = 0.087), respectively] (Table 2).

**Figure 1:**
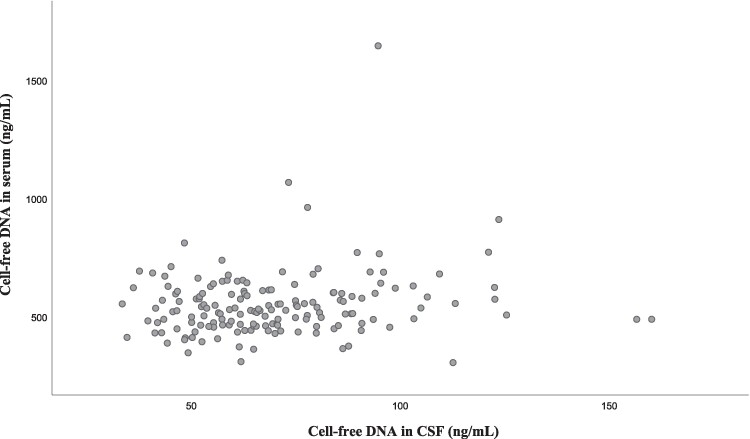
**Correlation between cell-free DNA in CSF and serum of hip fracture patients (*n* = 135).** Spearman's rho = 0.082, *P* = 0.343.

In CSF, only cell-free DNA was detectable in all samples. Despite several years in the freezer, there was no significant difference in CSF cell-free DNA levels between samples from the first and second hip fracture cohorts, respectively [68 (56, 85) ng/ml versus 67 (54, 84) ng/ml, *P* = 0.651]. Thus, all available CSF samples were analysed. The NET marker CitH_3_ was undetectable in most CSF samples (62%), whereas MPO-DNA was undetectable in all CSF samples. Concentrations of CitH_3_ and MPO-DNA in CSF were therefore not included in further statistical analyses. All NET markers were detectable in serum and were moderately inter-correlated (*r* = 0.286–0.471, *P* < 0.001, all). Cell-free DNA in serum and CSF did not correlate significantly (Fig. 1). It should be noted that the levels of cell-free DNA were ∼8-fold higher in serum than in CSF.

### Cell-free DNA and NET markers in hip fracture patients—stratified by dementia status

**Figure 2:**
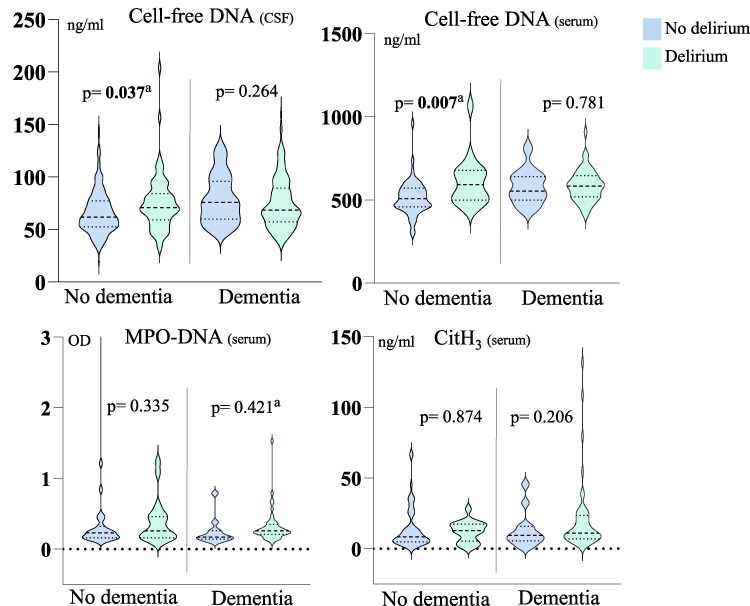
**Distribution of cell-free DNA and NET markers of hip fracture patients without versus with delirium, stratified for dementia status.** Median (bold dotted line) and 25 and 75 percentiles (dotted lines) are indicated in the figure. The Mann–Whitney U-test was used for group comparisons between the two groups ‘no delirium’ and ‘delirium’ in the no dementia and dementia group, separately. ^a^Adjusted for age and sex in multivariate binary logistic regression analyses. Sample size in the different groups is shown in Supplementary Table 2.

In patients without dementia (*n* = 289), cell-free DNA in CSF and serum was significantly higher in patients with versus without delirium [70 (59, 84) versus 62 (53, 77) ng/ml, *P* = 0.005) and (601 (504, 684) versus 508 (458, 572) ng/ml, *P* = 0.002] (Supplementary Table 2). These associations remained significant after adjusting for age and sex (Fig. 2 and Table 3). Serum MPO-DNA and CitH_3_ were both numerically higher, though not significantly different between the delirium groups (Supplementary Table 2).

**Table 3:** Association between the NETs marker cell-free DNA in CSF and serum and delirium in hip fracture patients without dementia

	Unadjusted				Adjusted^a^			
	β	*P*-value	OR	95% CI	β	*P*-value	OR	95% CI
Cell-free DNA in CSF	0.016	**0.007**	1.016	1.004–1.028	0.013	**0.037**	1.014	1.001–1.026
Cell-free DNA in serum	0.007	**0.012**	1.007	1.001–1.012	0.007	**0.007**	1.007	1.002–1.013

Univariate and multivariate binary logistic regression analyses.

NETs; neutrophil extracellular traps, OR; odds ratio, CI; confidence interval.

^a^Adjusted for age and sex. Significant *P*-values are highlighted with boldface.

In patients with dementia (*n* = 202), only serum MPO-DNA was significantly higher in patients with delirium versus no delirium (0.255 versus 0.170 optical density, *P* = 0.044) (Supplementary Table 2). However, the significance was lost after adjusting for age and sex (*P* = 0.421).

### Cell-free DNA and NET markers in delirium subgroups

**Figure 3:**
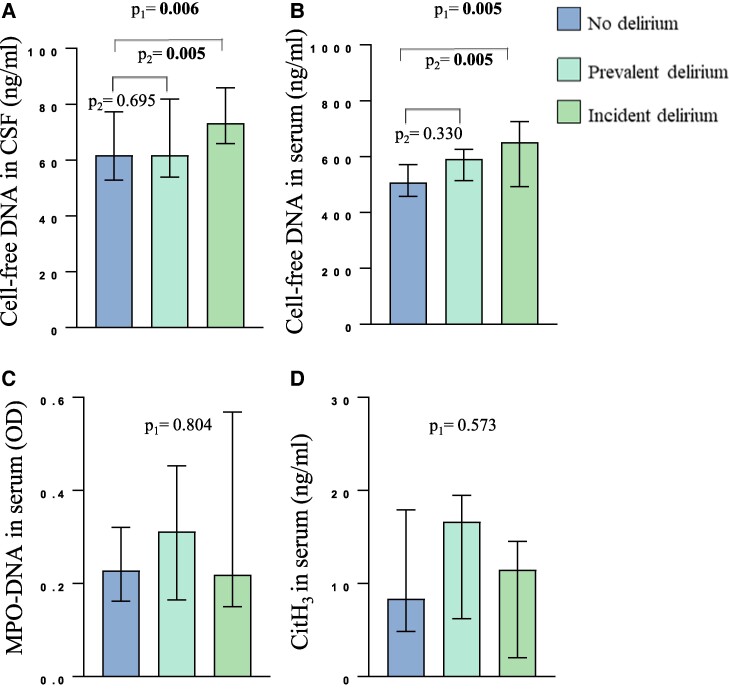
**Cell-free DNA and NET markers in CSF and serum of hip fracture patients without dementia according to delirium subgroups (*n* = 289).** (**A**) Cell-free DNA in CSF, (**B**) cell-free DNA in serum, (**C**) MPO-DNA in serum and (**D**) CitH_3_ in serum according to groups of delirium. Sub-syndromal delirium (SSD) (*n* = 26) is included in the no delirium group (total *n* = 221), prevalent delirium (*n* = 27) is defined as delirium diagnosed preoperatively, and incident delirium (*n* = 41) defined as delirium onset after CSF sampling. A one-way ANOVA is used in the between group comparisons of levels of cell-free DNA and NET markers for the delirium subgroups (p_1_-values). *Post hoc* comparisons using the Tukey's Honestly Significant Difference test is applied for pairwise comparisons (p_2_-values).

Cell-free DNA and NET markers according to subgroups of delirium, are presented in Fig. 3. In CSF, significantly higher levels of cell-free DNA were observed in incident delirium versus no delirium (*P* = 0.001). In serum, cell-free DNA levels were significantly higher in incident versus no delirium (*P* = 0.007), but not significantly higher in prevalent versus no delirium (*P* = 0.057) (details in Fig. 3). Sensitivity analyses with SSD patients (*n* = 26) excluded from the no delirium group did not significantly change our results. MPO-DNA and CitH_3_ did not differ significantly between the delirium subgroups.

### Correlation between CSF levels of cell-free DNA and NfL

**Figure 4:**
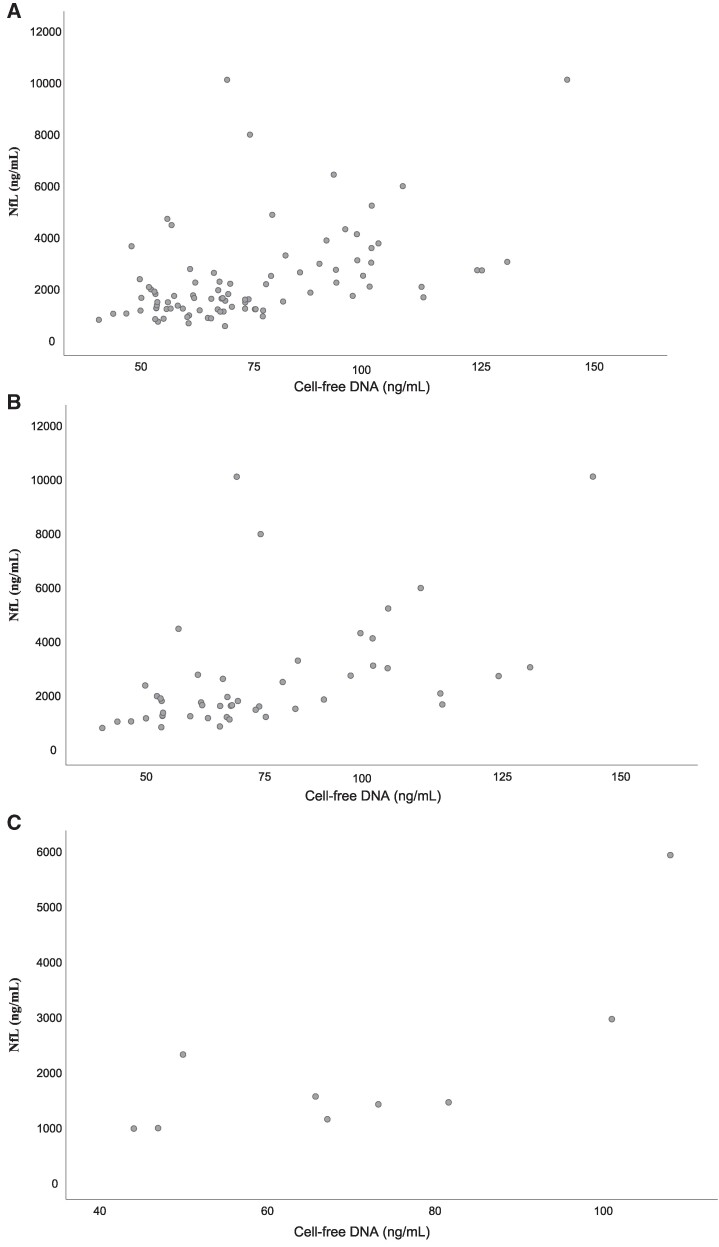
**Associations between cell-free DNA and NfL in CSF of hip fracture patients** (**A**) Hip fracture patients in general (*n* = 89): spearman's rho = 0.441, *P* < 0.001. (**B**) Hip fracture patients with delirium (*n* = 48): spearman's rho = 0.468, *P* < 0.001. (**C**) Hip fracture patients with delirium and without dementia (*n* = 9): spearman's rho = 0.765, *P* = 0.045. All adjusted for age and sex.

NfL analyses were available in 89 patients, and the associations with delirium have previously been reported.^29^ In the current study, CSF cell-free DNA correlated positively with NfL in hip fracture patients in general (spearman's rho = 0.441, *P* < 0.001) (Fig. 4A). The correlation was stronger in patients with delirium and strongest in delirious patients without dementia (spearman's rho = 0.468, *P* < 0.001 and 0.768, *P* = 0.045, respectively (Fig. 4B and C). All adjusted for age and sex.

## Discussion

The main finding of the present study was that delirious hip fracture patients without dementia had significantly higher levels of cell-free DNA in both serum and CSF. Hip fracture patients in general had significantly higher levels of cell-free DNA and NET markers in serum than healthy controls. We also showed an association between CSF levels of cell-free DNA and NfL, which may link cell-free DNA to neuronal damage in delirium, a relationship that should be explored in more detail in future studies.

### Cell-free DNA and NETs in serum and CSF of hip fracture patients versus healthy controls

Cell-free DNA, CitH_3_ and MPO-DNA were higher in serum of hip fracture patients than healthy controls. This is in line with recent data implying that NETs and cell-free DNA play a role in disorders with an inflammatory component such as trauma or atherosclerosis.^19,40^ Post injury, cytokines and inflammatory mediators, such as IL-1β, TNFα, IL-8 and damage-associated molecular patterns^41-43^ may stimulate neutrophils to change phenotype and promote tissue damage by releasing reactive oxygen species, NETs and cell-free DNA. Of note, CSF cell-free DNA was not significantly different between hip fracture patients and healthy controls.

### Cell-free DNA, NETs and delirium

The significant association found between cell-free DNA and delirium in both serum and CSF in those free of dementia may indicate that cell-free DNA-producing processes are implicated in delirium pathophysiology, meriting further investigation. However, the more specific NET markers CitH_3_ and MPO-DNA, only assessed in serum, were just numerically higher in these patients. Therefore, we cannot provide evidence of NETosis per se to be implicated in delirium. It might also be discussed whether increased cell-free DNA could be due to increased systemic inflammation that subsequently develops into delirium. Consistent with our hypothesis and results, a recent study exploring the compartmental source of extracellular DNA during systemic inflammation found that cell-free DNA predominantly derived from neutrophils during low-grade endotoxaemia in healthy humans compared to non-neutrophil sources in severe systemic inflammation caused by sepsis.^44^

In the systemic circulation, cell-free DNA and proteases have a direct toxic effect on the endothelial cells and may contribute to a chronic inflammatory milieu and increased vascular permeability.^45^ A recent report showed that biomarkers of endothelial activation and neurological injury (S100 calcium binding protein B) in plasma were associated with prolonged delirium in patients hospitalized with organ failure.^46^ In another study, the NET components neutrophil elastase and cathepsin G were shown to mediate BBB disruption by degrading basement membrane laminins.^47^ BBB damage and transmigration of neutrophils and other immune cells into the CNS are hallmarks of neuroinflammation.^48^ Allen *et al*.^20^ have previously shown that neutrophils localize with neurons and decrease their viability in vitro. Additionally, circulating neutrophils with a hyper reactive phenotype have been shown to correlate with the rate of cognitive decline in Alzheimer's disease.^23^ Interestingly, treatment with kynurenic acid, a neuroprotective metabolite in the kynunerine pathway, in rats with sepsis showed reduced BBB injury, reduced brain mitochondrial dysfunction and reduced NET formation (CitH_3_ and MPO).^49^ In the present study, levels of cell-free DNA in serum and CSF were not inter-correlated. This supports a restrictive BBB rather than direct exchange of cell-free DNA through the BBB. Cell-free DNA in the CSF may be a result of NETosis or other types of cell death and damage, and therefore we cannot conclude whether it represents an evidence of netting neutrophils. Lower levels of the NET markers MPO-DNA and CitH_3_ were expected in CSF versus in serum. Unfortunately, these markers were undetectable in CSF, which may be due to the low sensitivity of the methods or different cellular stimuli and release of the individual components. In the subgroups of delirium, our study showed that cell-free DNA was significantly higher in hip fracture patients developing delirium after surgery (incident delirium) than in patients with no delirium. Cell-free DNA has been shown to activate innate immune cells, and may explain the parallel increase of the myeloid derived inflammatory cytokine IL-8, previously shown in the same patient cohort.^10^ As cell-free DNA has a short half-life in blood, it might represent a sensitive and real-time predictor of disease development.

### Cell-free DNA, NETs and the interplay between delirium and dementia

In patients with established dementia, cell-free DNA and NET marker levels did not differ between patients with and without delirium. This observation may be explained by the fact that patients with dementia are more vulnerable to delirium and might develop delirium without a parallel increase in inflammation.^4^ This is also in line with our previous report of other inflammatory markers that associated with delirium only in those free of dementia.^9^ Moreover, a significant association between the plasma concentration of IL-8 and the duration of delirium have been found in patients without clinical dementia.^50^ In contrast to our results, neutrophils and NETs have been shown in the vasculature and parenchyma of individuals with Alzheimer's disease, the most common form of dementia.^21^ Different methodological approaches in diagnosing dementia and uneven patient population characteristics might explain these contrasting results. The patients in our study were relatively old, and diminished NET release along increasing age due to less reactive oxygen species production and defective autophagy has been reported.^51,52^

### Cell-free DNA and the neuronal damage marker NfL

We found a strong correlation between CSF levels of cell-free DNA and NfL, which suggests neuronal damage and release of cell-free DNA are present during delirium. Whether this finding represents NET-induced injury of neurons or simply is a result of delirium induced injury of neurons and glial cells with release of their DNA, remains to be answered. As part of future work, it would be interesting to measure NET markers with more sensitive methods, simultaneously with NfL in both CSF and blood at several time points. NETs in the brain parenchyma may proteolyse extracellular matrix proteins and activate inflammasome- and mitochondrial apoptotic pathways with harmful effect on nerve cells.^21,22,53^ The association we found between cell-free DNA and NfL could be discussed as a pathophysiological mechanism in delirium being a strong risk factor for future dementia.^54^

### Strengths and limitations

There are several strengths in our study, including novelty, large sample size and the inclusion of a cognitively healthy control group. A subset of the patients had CSF and blood samples drawn at the same time, allowing for direct comparisons between the two compartments. Unfortunately, MPO-DNA and CitH_3_ were not detectable in most of the CSF samples. It would have been valuable to measure these biomarkers over multiple time points to observe fluctuations. However, the collection of CSF, which more accurately represents processes occurring in the brain, holds ethical and practical challenges, especially in people with delirium and repeated samplings of CSF is therefore hard to do. The samples used in the present study originated from two hip fracture cohorts sampled across non-overlapping periods; however, including cohort as a variable in the multivariate statistical models did not change the results. Age is a well-known factor for development of delirium and dementia, and as our cognitively healthy control group was younger, this may have influenced our results. For future work, neutrophil counts should be included, as well as taking other comorbidities that might affect cell-free DNA and NET concentration, such as heart disease,^55-57^ visceral obesity, and hyperlipidaemia into account.^58^ Due to the explorative nature of the study, our results needs to be confirmed in a future trial.

In conclusion, the current study shows significantly higher levels of cell-free DNA in blood and CSF of delirious hip fracture patients free of dementia. This may imply that cell-free DNA takes part in delirium development, potentially mediated via neuronal damage, which is supported by the association between cell-free DNA and NfL.

## Supplementary Material

fcae452_Supplementary_Data

## Data Availability

The data supporting the findings of this research article are present within the article and/or the Supplementary Materials. The original dataset is available from the corresponding author, upon reasonable request.
