# Supplementary material for: Increased cell-free DNA in CSF and serum of hip fracture patients with delirium
Source: Brain Commun. 2024 Dec 16;7(1):fcae452. doi: 10.1093/braincomms/fcae452 (PMC11683831; doi:10.1093/braincomms/fcae452)
Supplement: fcae452_Supplementary_Data [file fcae452_supplementary_data.pdf]

Supplementary Table 1. Overview of available sample material in hip fracture patients and cognitively healthy controls

**Sample material**	**Cohorts**	
	Hip fracture patients	*Cognitively healthy controls*
CSF (n= 523)	n= 491	n= 32
Serum (n= 161)	n= 135	n= 26

Supplementary Table 2. Levels of NET markers in CSF and serum of hip fracture patients without and with delirium, stratified for dementia status

		CSF	Serum		
		Cell-free DNA (ng/mL)	*Cell-free DNA (ng/mL)*	*MPO-DNA (OD)*	*$CitH_3$ (ng/mL)*
**No dementia** (n= 289)	No delirium (n= 221)	62 (53, 77)	508 (458, 572) n= 55	0.228 (0.162, 0.321) n= 53	8.37 (4.85, 17.92) n= 54
	Delirium (n= 68)	70 (59, 84)	601 (504, 684) n= 20	0.257 (0.159, 0.456) n= 20	12.31 (4.39, 17.20) n= 20
	p-value[a]	**0.005**	**0.002**	0.335	0.874
**Dementia** (n= 202)	No delirium (n= 42)	76 (60, 96)	554 (499, 640) n= 13	0.170 (0.145, 0.263) n= 12	9.55 (5.54, 15.71) n= 13
	Delirium (n= 160)	68 (57, 89)	583 (519, 647) n= 47	0.255 (0.207, 0.349) n= 45	10.96 (7.07, 23.45) n= 47
	p-value[a]	0.264	0.781	**0.044**	0.206

[a] The p-value reflects whether there is a statistical significant difference in NET markers between the no delirium and delirium subgroup (Mann Whitney *U* test). Significant p-values are highlighted with boldface. The subsyndromal delirium (SSD) (n= 26) subgroup is a part of the no delirium group (n= 221).

Abbreviations: MPO-DNA; myeloperoxidase-DNA, $CitH_3$; citrullinated histone H_3
